# The Dolenc technique was used to clip 14 cases of ruptured basilar apex aneurysms and posterior cerebral artery aneurysms

**DOI:** 10.3389/fneur.2022.928676

**Published:** 2022-07-26

**Authors:** Zhang Hongwei, Xie Kang, Li Aimin, Zhang Dong

**Affiliations:** ^1^Department of Neurosurgery, Beijing Tiantan Hospital Affiliated to Capital Medical University, Beijing, China; ^2^Department of Neurosurgery, Lianyungang Hospital Affiliated to Xuzhou Medical University, Lianyungang, China

**Keywords:** Dolenc approach, surgical clipping, basilar apex aneurysm, posterior cerebral artery aneurysm, Rankin scale scores

## Abstract

**Objective:**

To investigate the surgical techniques and their clinical effects for ruptured basilar artery apex and posterior cerebral aneurysms *via* the Dolenc approach.

**Methods:**

We retrospectively analyzed the clinical data of 14 patients with ruptured basilar artery apex and posterior cerebral aneurysms who underwent surgical clipping by the Dolenc approach from July 2017 to June 2020 in Beijing Tiantan Hospital affiliated with Capital Medical University and Lianyungang Hospital affiliated with Xuzhou Medical University. The modified Rankin scale (mRs) scores were used to evaluate the prognosis of patients.

**Results:**

All 14 cases of aneurysms were successfully clipped. Overall, 1, 2, and 1 cases of postoperative new-onset visual loss, oculomotor nerve palsy, and contralateral hemiplegia, respectively, were reported. Digital subtraction angiography (DSA) or computed tomography angiography (CTA) examination of the aneurysm within 2 weeks after surgery revealed that the aneurysm was completely clipped without residue. The observations during the follow-up within 12–18 months after surgery were as follows: 1 case of vision loss returned to normal, 2 cases of oculomotor nerve palsy, 1 case of return to normal, 1 case of improved, 1 case of contralateral hemiplegia improved after rehabilitation treatment, and 1 case of hydrocephalus ventriculoperitoneal shunt surgery was performed. Overall, 11, 2, and 1 case had mRs scores of 0, 1, and 2, respectively. No death was reported.

**Conclusion:**

For the small number of basilar apical and posterior cerebral aneurysms treated non-invasively, the Dolenc approach may offer advantages over other modalities.

## Introduction

Apical basilar artery aneurysms and posterior cerebral artery aneurysms are relatively rare intracranial aneurysms, accounting for ~2–7 and 1–2% of intracranial aneurysms, respectively (1, 2). Due to the deep location and complex surrounding anatomy, the treatment of intracranial aneurysms is challenging for neurosurgeons and neurointerventionalists. Currently, interventional therapy is the main treatment for basilar artery apical aneurysms and posterior cerebral aneurysms. However, because of the advantages of craniotomy clipping in terms of the complete occlusion rate and recurrence rate, craniotomy clipping is still an irreplaceable choice.

The Dolenc approach is one of the most commonly used surgical approaches for the surgical treatment of basilar artery apical aneurysms. It was first proposed by Professor Vinko Dolenc in 1987, who applied it to 11 cases of basilar artery apical aneurysms with good results. This approach features epidural resection of the anterior clinoid process combined with subdural anterior access through the cavernous sinus into the interpeduncular cistern to expose and clip the basilar aneurysm (3–5). Moreover, the Dolenc approach has certain advantages in the surgical treatment of posterior cerebral aneurysms.

In this study, we retrospectively analyzed the clinical outcomes of 14 patients with apical basilar artery or posterior cerebral aneurysms clipped by the Dolenc approach and analyzed the feasibility, safety, and advantages of the Dolenc approach in the treatment of such aneurysms.

## Materials and methods

### General information

In this study, 14 patients (8 with basilar artery apex and 6 with posterior cerebral aneurysms) were included. Of the 8 patients with basilar artery apex aneurysms, 3 were men and 5 were women, with an average age of 59.4 (range: 39–72) years. All eight patients had a sudden headache as the first symptom. According to the Hunt–Hess classification score, 1, 5, 1, and 1 cases were grades I, II, III, and IV, respectively. The six cases of posterior cerebral aneurysms included 4 men and 2 women, with an average age of 52.8 (range 33–64) years. All of them had a sudden headache at the time of onset, and 2 of them presented with a complication of oculomotor nerve palsy. According to the Hunt–Hess classification, 2, 1, 1, 1, and 1 cases were grades I, II, III, IV, and V, respectively.

### Imaging examination

Overall, 14 cases were diagnosed with posterior circulation aneurysm using digital subtraction angiography (DSA) and computed tomography angiography (CTA). Among them, 8 cases were of basilar artery apical aneurysm, of which 7 aneurysms were located at the origin of the P1 segment of the posterior cerebral artery and 1 was located at the origin of the superior cerebellar artery. Among the 8 cases of basilar aneurysm, 6 cases were combined with other aneurysms. Considering the size of the aneurysm, 3, 4, and 1 case had a diameter <0.5 cm, ≥ 0.5 but <1.5 cm, and ≥ 1.5 but <2.5 cm, respectively. Among 6 cases of the posterior cerebral aneurysm, 2, 3, and 1 were located in the P1 segment of the posterior cerebral artery, at the P1–P2 bifurcation, and in the P2 segment, respectively. Considering the size of the aneurysm, 1, 4, and 1 case had a diameter <0.5 cm, ≥ 0.5 but <1.5 cm, and ≥ 1.5 but <2.5 cm, respectively.

### Surgical methods

After general anesthesia, the patients were placed in the supine position. A standard pterional craniotomy was used for the surgical approach; the bone flap was removed, a part of the sphenoid ridge was bitten off, and the dura was further separated from the anterior clinoid process. A part of the upper lateral wall of the optic canal was removed, and a part of the anterior clinoid process, orbital roof, and lesser sphenoid wing were further removed. The whole process of micro-drilling drips water to ensure timely cleaning, and when the air chamber is opened, the mucosa should be kept intact and the bone wax should be closed as much as possible. When peeling the dura, the bleeding of the cavernous sinus should be stopped by compression with a gelatin sponge. Further, the dura mater was cut along the “T” shape of the lateral fissure, the suture was pulled on both sides, and the arachnoid cistern was opened to release the cerebrospinal fluid. The optic nerve dural sheath along with the proximal and distal rings of the internal carotid artery was opened, and the internal carotid artery was released. The lateral space of the internal carotid artery was opened and fully removed the attachments around the posterior communicating artery, exploring the posterior communicating artery to the P1-P2 bifurcation of the posterior cerebral artery, and then proceeded to the proximal end of P1, which can be exposed to the top of the basilar artery or to the distal end of P2. Exposure, such as the posterior clinoid process affecting the aneurysm or exposure of the proximal parent artery, may be removed.

## Results

### Clinical efficacy

All 14 cases of aneurysm were successfully clipped during the surgery, 2 cases of aneurysm ruptured and hemorrhaged during surgery, among which, 1 case was a basilar aneurysm, no temporary closure was performed, the other case was a posterior cerebral aneurysm with a large aneurysm, and the proximal parent artery was temporarily blocked and then clipped. One case of the basilar aneurysm was observed to be combined with the left posterior communicating aneurysm and anterior communicating aneurysm by preoperative DSA. The posterior communicating artery ampulla was detected during the surgery; no anterior communicating aneurysm was observed, and the basilar aneurysm was successfully clipped. In the early postoperative period, visual acuity decreased in 1 case, new ophthalmic nerve palsy was observed in 2 cases, and contralateral hemiplegia was observed in 1 case. The DSA or CTA examination of the aneurysm within 2 weeks after the surgery revealed that the aneurysm was completely clipped without residue. During a follow-up of 12–18 months after the surgery, 1 case of vision loss returned to normal; among the 2 cases of oculomotor nerve palsy, 1 returned to normal, and 1 case was improved. One case underwent ventriculoperitoneal shunt surgery because of hydrocephalus. According to the modified Rankin scale (mRs) prognostic score, the prognosis of patients with aneurysms was evaluated. There were 11 cases with mRs 0, 2 with mRs 1, and 1 with mRs 2. No death was reported.

**Figure 1:**
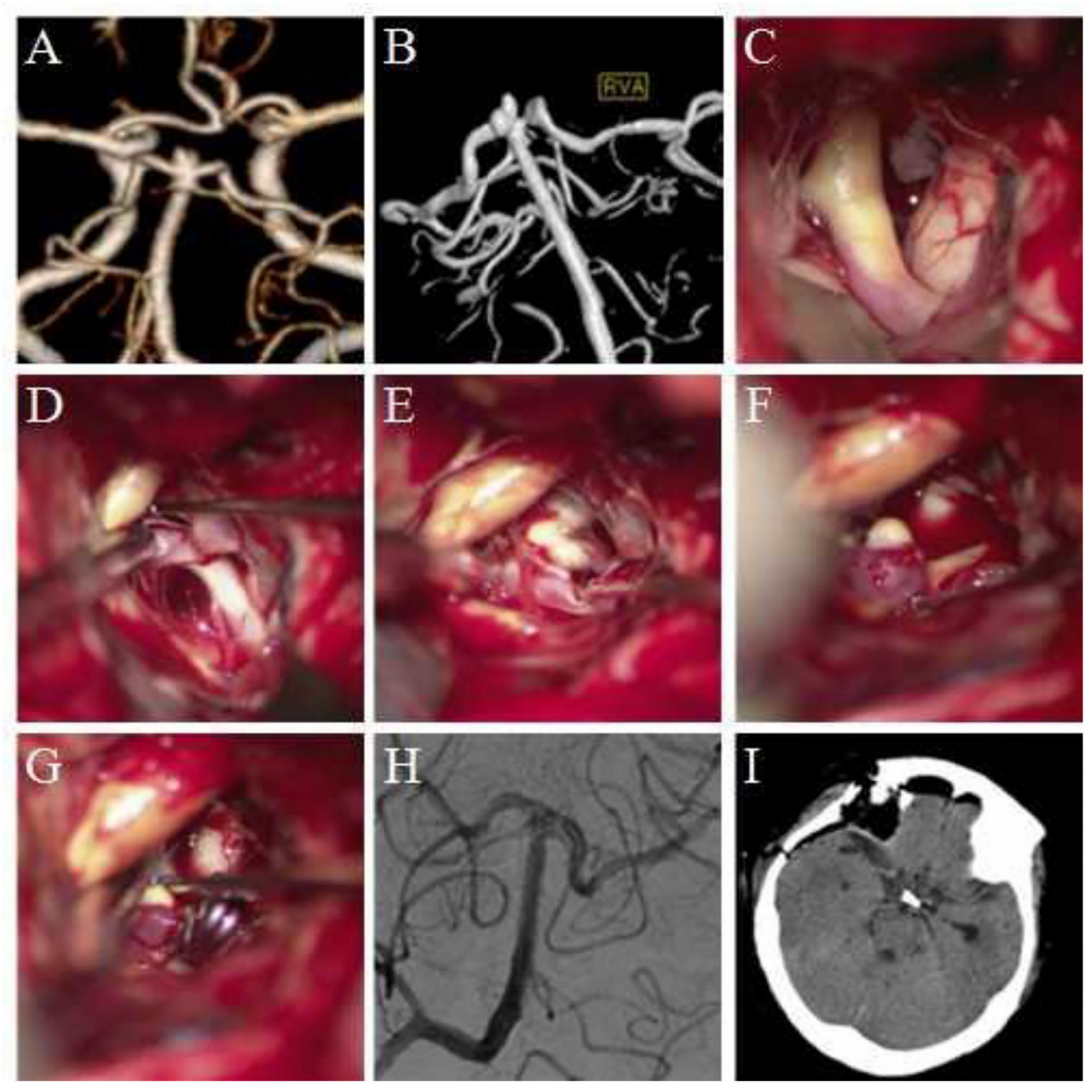
Clipping of basilar artery apical aneurysm *via* the Dolenc approach. **(A,B)** Preoperative computed tomography angiography (CTA) and digital subtraction angiography (DSA) revealed a basilar artery apical aneurysm; the aneurysm was lobulated. **(C)** During surgery, the internal carotid artery and the posterior clinoid process of the surgery side were exposed. **(D)** The internal carotid artery was pulled medially, and the exposure range was increased. The P1 segment of the posterior cerebral artery and its perforating arteries were visible. **(E)** The basilar artery was exposed along the posterior cerebral artery to the top of the basilar artery. The aneurysm was exposed, and the sub-aneurysm was visible. **(F)** The aneurysm was completely exposed, and sub-aneurysms were visible. **(G)** The neck of the aneurysm was clipped. The basilar arteries, bilateral posterior cerebral arteries, and superior cerebellar arteries were explored, and no vascular damage was observed. **(H)** Intraoperative DSA revealed no development of aneurysm and no vascular damage. **(I)** Re-examination was performed using the head CT within 12 h after surgery.

**Figure 2:**
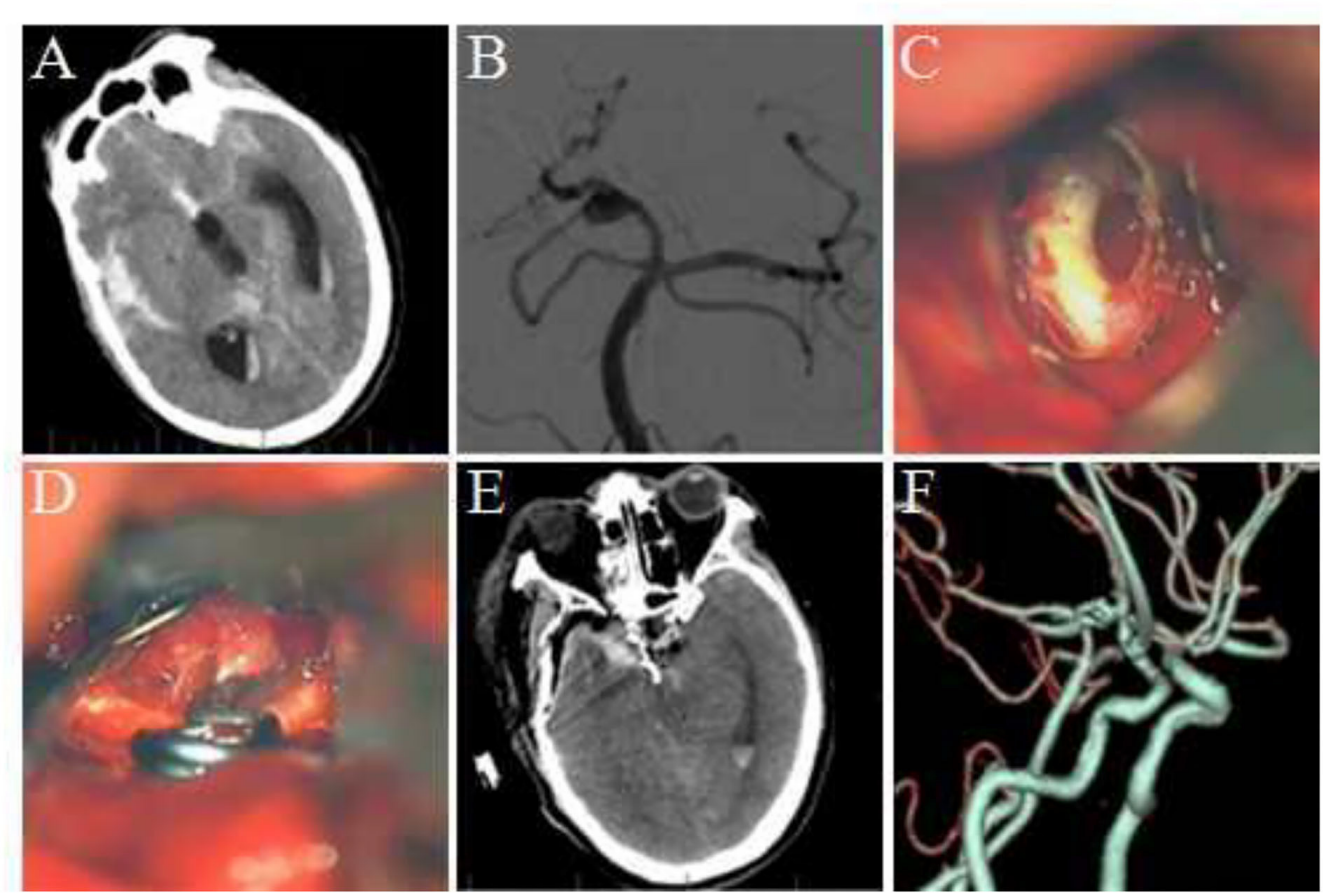
Clipping of the aneurysm at the P1–P2 bifurcation of the posterior cerebral artery *via* the Dolenc approach. **(A)** Preoperative head CT revealed subarachnoid hemorrhage. **(B)** DSA revealed the tumor at the P1–P2 bifurcation of the right posterior cerebral artery. **(C)** The aneurysm was exposed during the surgery; the wall of the apical aneurysm was thin, and adhesion was observed between the aneurysm and temporal lobe. **(D)** The aneurysm was clipped parallel to the parent artery during the surgery. **(E)** Re-examination using head CT within 12 h after surgery. **(F)** Postoperative CTA revealed no development of aneurysm and no vascular damage.

**Figure 3:**
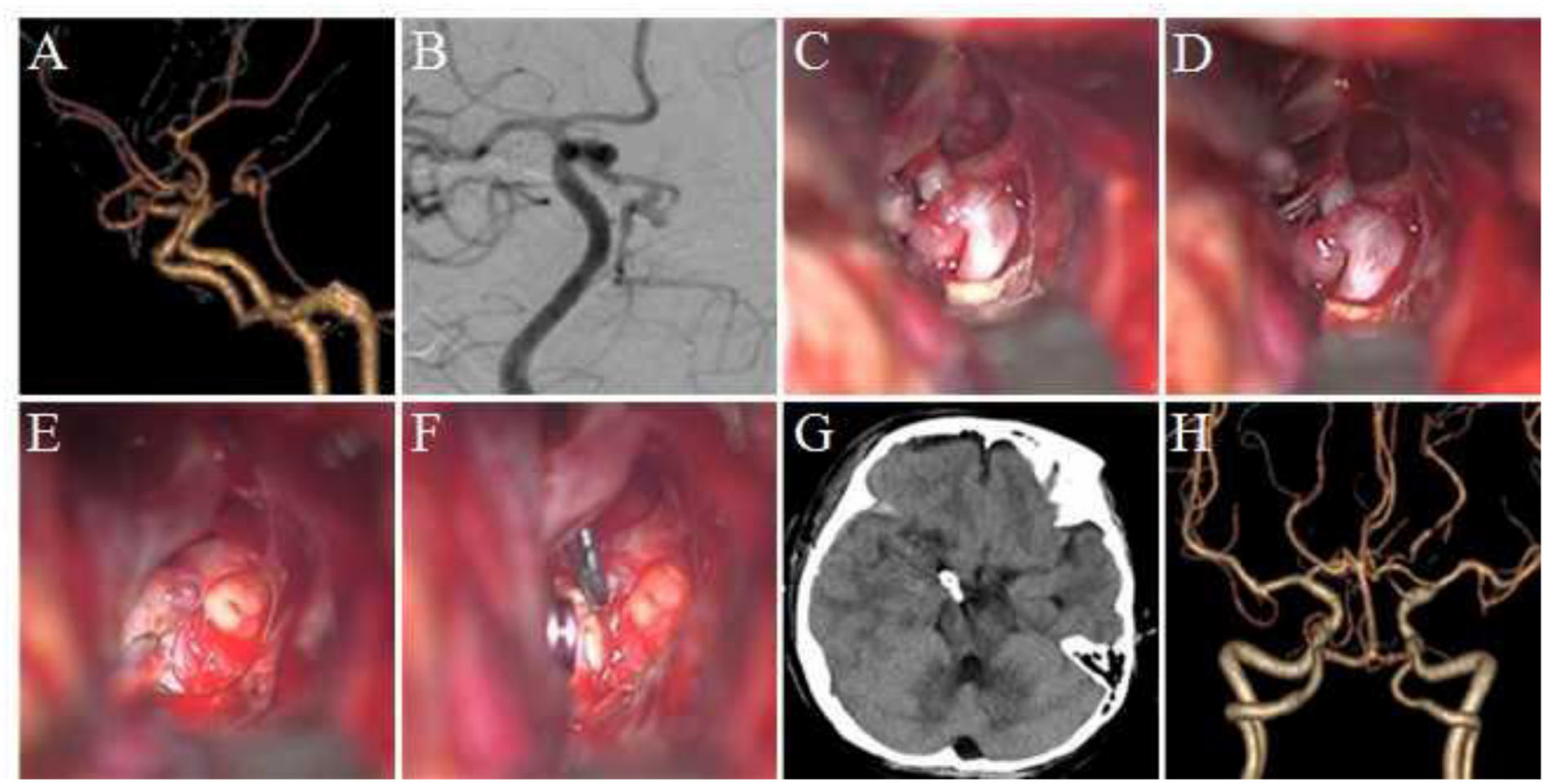
Clipping multiple aneurysms at the P1–P2 bifurcation of posterior cerebral aneurysm *via* the Dolenc approach. **(A)** Preoperative CTA revealed an aneurysm at the left posterior cerebral P1–P2 bifurcation with irregular shape and basilar artery and bilateral posterior cerebral artery stenosis. **(B)** DSA revealed two aneurysms at the bifurcation of P1–P2 of the posterior cerebral artery, exhibiting a lobulated shape. **(C)** During surgery, the P1–P2 bifurcation aneurysm could be seen in the lateral space of the internal carotid artery, which points to the posterior and medial side, and the oculomotor nerve and posterior communicating artery could be seen. **(D)** The optic nerve was pulled medially to increase the exposure range, and the P1 segment of the posterior cerebral artery, posterior communicating artery, and its perforating vessels could be seen. **(E)** Another aneurysm at the bifurcation of P1–P2 was exposed, pointing downward, and a part of the perforating vessels and oculomotor nerve from P2 could be seen. **(F)** Two aneurysm clips were used to clip the aneurysm. **(G)** A postoperative CT scan of the head. **(H)** The postoperative CTA revealed that the aneurysm was completely clipped with no residue and no vascular damage.

Considering the location, shape, size, and other factors of the aneurysm, we selected 3 typical cases: (1) Case 1: A 72-year- old female was admitted to the hospital because of a sudden headache for 2 weeks. Preoperative CTA and DSA examination revealed a lobulated basilar artery apical aneurysm. The right Dolenc approach aneurysm clipping was performed, and the aneurysm was exposed and clipped successfully during the surgery. (2) Case 2: A 55–year-old male was admitted to the hospital because of a sudden severe headache with unconsciousness for 2 days. Preoperative DSA revealed an aneurysm at the P1–P2 bifurcation of the right posterior cerebral artery. The right Dolenc approach was performed for aneurysm clipping. During the surgery, the aneurysm was seen to be blood blister-like. The proximal P1 segment was temporarily blocked and the aneurysm was clipped. (3) Case 3: A-56-year old female was admitted to the hospital because of a sudden headache. Preoperative CTA and DSA revealed multiple aneurysms at the P1–P2 bifurcation of the right posterior cerebral artery. The right aneurysm was clipped *via* the Dolenc approach. The two aneurysms were exposed and clipped smoothly during the surgery. The imaging data and intraoperative findings of typical cases before and after surgery are shown in Figures 1–3.

## Discussion

The basilar artery and the posterior cerebral artery are one of the most common sites for the occurrence of posterior circulation aneurysms, among which the top of the basilar artery is the most common site (6, 7). Since aneurysm of the posterior circulation continues to grow and has a high risk of rupture, most of them require active surgical treatment. The top area of the basilar artery contains many branched vessels with important functions and perforating arteries to the brainstem and thalamus. Hence, interventional therapy and surgical clipping are difficult and risky. A large number of posterior circulation aneurysms are selected for interventional therapy. However, surgical clipping of aneurysms is still a good choice, particularly, for complex aneurysms, such as those at complex morphological structures, recurrent aneurysms after the intervention, those where branch vessels are closely related, and those that are large or huge, wide-necked, calcified, or thrombosed. Moreover, surgical clipping has certain advantages (8). Studies have compared the therapeutic effects of interventional therapy and surgical clipping in 208 cases of basilar artery apical aneurysms. No significant difference was observed in the terms of prognosis. However, the recurrence rate of surgical clipping was lower than that of interventional therapy (9). A study reported that surgical clipping is superior to interventional therapy in terms of complete occlusion rate, rebleeding, and retreatment for the treatment of basilar artery apical aneurysms, and surgical clipping is more suitable for young patients (10). The cases chosen in this study for craniotomy and clipping were mainly based on the following points: (1) the interventional doctor believed that interventional therapy was not suitable because of the shape of the aneurysm. (2) There were indications for craniotomy and clipping surgery. (3) Patients and their families chose surgery because of cost and other reasons.

Common surgical approaches for basilar aneurysms and posterior cerebral aneurysms include pterional and infratemporal approaches, both of which have their own advantages and disadvantages (11, 12). The pterional approach is simple to operate. However, the space is relatively small, the visual field is easily blocked by the internal carotid and posterior communicating arteries, and the proximal basilar artery is poorly exposed. Particularly, the basilar artery top is located low or the aneurysm points to the rear. The Sylvian fissure should be fully opened and the temporal lobe should be stretched. The pterional approach can satisfactorily expose the P1–P2 bifurcation of the posterior cerebral artery. The subtemporal approach can effectively shorten the surgical path, and the main trunk of the basilar artery is well exposed. The exposure of the perforating artery is difficult, the temporal lobe is greatly stretched, and the cranial nerve is easily damaged. Studies have reported that the incidence of oculomotor nerve injury in patients undergoing subtemporal approach surgery is 32–35%. It is mainly manifested as postoperative oculomotor nerve palsy, of which 8–9% cases are difficult to recover (13). The inferior temporal approach has more advantages in the exposure of the P2 segment of the posterior cerebral artery.

The posterior cerebral artery arises from the top of the basilar artery, and the aneurysm at the tip of the basilar artery is often exposed proximally along the posterior cerebral artery with the Dolenc approach. The Dolenc approach has certain advantages in the treatment of basilar artery and posterior cerebral artery aneurysms: (1) the anterior clinoid process is removed by epidural grinding. The distal and proximal rings of the internal carotid artery and the optic nerve sheath are opened. The internal carotid artery and the optic nerve are released, and their mobility is improved. The exposed area of the anatomical space related to surgery is increased, such as the optic nerve–internal carotid triangle, internal carotid artery–oculomotor triangle, and the oculomotor–tentorial triangle. (2) The resection of the anterior clinoid process moves the surgical perspective forward, which can better use the natural intracranial space, avoid excessive temporal lobe, wide open the Sylvian fissure and reduce damage. (3) The increase of the operating space and the change of the surgical angle are beneficial to the exposure of the top of the basilar artery, and the clipping of high, normal, and low basilar artery apical aneurysms. At the same time, the P1 segment of the contralateral posterior cerebral artery and the superior cerebellar artery can be exposed. (4) Increased exposure to posterior cerebral arteries. (5) It is beneficial for the temporary block of the parent artery and protection of the cranial nerve during the surgery, and it improves the safety of surgery. (6) It is beneficial in the clipping of special cases of large or huge, blood blister-like, or irregular aneurysms and intraoperative aneurysm rupture. (7) If necessary, the clinoid process can be further exposed after grinding. Some studies have measured the exposure range of the basilar artery by simulating the orbital zygomatic approach through the cavernous sinus through cadaveric head dissection. The average length of exposure is 4 mm, and after the anterior clinoid process is removed, the exposure length is 16.5 mm, and the exposure length is significantly increased (14). In addition, some studies believe that the fronto-orbitozygomatic approach can simultaneously remove the anterior and posterior clinoid processes and treat basilar artery apical aneurysms at the optic nerve–internal carotid triangle, internal carotid artery–oculomotor triangle, and oculomotor–tentorial triangle. The optimal exposure and surgical approach to this site can be achieved; however, it is noted that this approach is relatively invasive (15).

Apical aneurysm of the basilar artery was clipped through the Dolenc approach, and mobility of the internal carotid artery was increased so that the internal carotid artery–oculomotor nerve space was the best exposure and surgery space. Since most aneurysms have wide necks and are located at bifurcations with many surrounding branch vessels, incomplete clipping of the aneurysm or misclamping of branch vessels is possible. Incorrect clipping and loss of the lateral posterior cerebral and superior cerebellar arteries and special attention should be paid to the protection of the perforating arteries to the brainstem and thalamus. If an aneurysm is irregular in shape, large in size, and has a blood blister-like shape that may rupture during surgery or fuse with surrounding branch vessels, the parent artery should be temporarily blocked, and then exposed and clipped to prevent intraoperative aneurysm and damage to blood vessels and nerves. In this study, 8 cases of basilar aneurysm were successfully clipped using the Dolenc approach, and 1 case exhibited rupture and hemorrhage during surgery. After suction, the neck of the aneurysm was clearly exposed and successfully clipped. In 1 case, the superior cerebellar artery originated from the aneurysm, and it was difficult to intervene, so surgery was chosen. The intraoperative aneurysm neck and basilar artery, posterior cerebral artery, and superior cerebellar artery were clearly exposed to clip successfully.

A posterior cerebral aneurysm is a relatively rare posterior circulation aneurysm, accounting for ~7% of posterior circulation aneurysms. It mostly occurs in the P1 segment, P1–P2 junction, and P2 segment of the posterior cerebral artery (16). Some studies have calculated the location of posterior cerebral aneurysms, such as the P1 segment, P1–P2 junction, and the P2-segment aneurysms, each accounting for 30%, and the P3 segment aneurysms accounting for 10% (17). After treatment of posterior cerebral artery aneurysm using the Dolenc approach, better exposure and viewing angle can be obtained, and the degree of freedom of surgery can be improved. During surgery, the posterior cerebral artery P1–P2 bifurcation can be exposed along with the origin of the internal carotid artery to the distal end of the posterior communicating artery, and then the P1 and P2 segments of the posterior cerebral artery can be exposed on both sides. It should be noted that traction of the blood vessel causes displacement of the parent artery, which leads to rupture of the aneurysm. The oculomotor nerve is located below the posterior cerebral artery, crosses with it, and is closely related. In this study, 2 cases of posterior cerebral aneurysm exhibited symptoms of oculomotor nerve palsy before surgery. Excessive pulling of the oculomotor nerve should be avoided during surgery of the tentorial triangle to prevent postoperative oculomotor nerve palsy symptoms. During surgery of the posterior cerebral artery P2-segment aneurysm, if it is difficult or impossible to clip the aneurysm, parent artery occlusion should be considered. In most cases, postoperative neurological dysfunction will not be caused. In this study, 6 cases of posterior cerebral aneurysms were clipped through the Dolenc approach. Among them, 1 case of the P1-segment aneurysm exhibited rupture and hemorrhage during surgery. Due to the large size of the aneurysm, the proximal end of the parent artery was temporarily blocked, and the aneurysm's neck and oculomotor nerve were exposed. After clearing, it was clipped smoothly.

Epidural resection of the anterior clinoid process is one of the key steps in the treatment of basilar artery apex and posterior cerebral aneurysm by the Dolenc approach. During the process, attention should be paid to the mechanical and thermal damage of the optic and oculomotor nerves, and to avoid postoperative cerebrospinal fluid nose caused by air chamber opening. Leakage, reasonable treatment of cavernous sinus hemorrhage, and dura mater along the Sylvian fissure “T” can reach the distal ring of the internal carotid artery so that the dural incision is reduced. Reasonable surgery of epidural is an important link to reduce postoperative complications. Oculomotor nerve palsy is a common postoperative complication of Dolenc's approach for such posterior circulation aneurysms. During surgery, the oculomotor nerve suffers from traction or bipolar thermal injury, or damage is observed to the perforating arteries from the posterior communicating artery and posterior cerebral artery that supply blood (18). At the same time, temporary blocking time should be reduced to prevent damage to perforating vessels and to reduce the occurrence of postoperative cerebral infarction. This is to particularly protect the perforating arteries of the brainstem and thalamus. Moreover, effective intraoperative multimodal combined monitoring, such as electrophysiological monitoring, intraoperative indocyanine green fluorescence angiography, DSA, and endoscopic exploration, can reduce the incidence of intraoperative vascular injury and postoperative cerebral ischemia.

Basal trunk aneurysms present a particular challenge to neurosurgeons due to high mortality and operative morbidity (19, 20). Different surgical methods result in poor prognosis of patients. Therefore, it is necessary to select appropriate surgical methods for different patients.

This study has certain limitations. The number of cases was lower, and it was a single-center study. Surgeons with varied experience often bring different treatment effects. In the future, studies including a high number of cases should be considered, and a prospective randomized controlled study should be conducted.

## Conclusion

Interventional therapy is the first-choice treatment for aneurysms that are present at the tip of the basilar artery and posterior cerebral aneurysms. However, for a few cases that are unacceptable to interventional physicians, craniotomy and clipping can be chosen. The Dolenc approach has more advantages than other surgical approaches. Therefore, the Dolenc approach is a safe and effective method for the surgical treatment of basilar artery apex and posterior cerebral aneurysms.

## Data availability statement

The original contributions presented in the study are included in the article/supplementary material, further inquiries can be directed to the corresponding authors.

## Ethics statement

The studies involving human participants were reviewed and approved by Beijing Tiantan Hospital Affiliated to Capital Medical University. The patients/participants provided their written informed consent to participate in this study. Written informed consent was obtained from the individual(s) for the publication of any potentially identifiable images or data included in this article.

## Author contributions

ZH: data analysis and interpretation and drafting of the manuscript. XK: acquisition of data and drafting of the manuscript. LA: study concept and design and critical revision of the manuscript. ZD: study concept and design and study supervision. All authors have read and approved the final manuscript.

## Funding

This work was supported by the Organization Department of Jiangsu Province and the Jiangsu Provincial Health Commission (ZDA20200181).

## Conflict of Interest

The authors declare that the research was conducted in the absence of any commercial or financial relationships that could be construed as a potential conflict of interest.

## Publisher's Note

All claims expressed in this article are solely those of the authors and do not necessarily represent those of their affiliated organizations, or those of the publisher, the editors and the reviewers. Any product that may be evaluated in this article, or claim that may be made by its manufacturer, is not guaranteed or endorsed by the publisher.

## References

[1] Li S Wang S Zhao YL Zhang D Zhao JZ. [Clinical characteristics and surgical outcomes of intracranial aneurysm: a retrospective study of 3322 cases]. Zhonghua Yi Xue Za Zhi. (2011) 91:3346–9. 22333202

[2] Brisman JL Song JK Newell DW. Cerebral aneurysms. N Engl J Med. (2006) 355:928–39. 10.1056/NEJMra052760 16943405

[3] Dolenc V. Direct microsurgical repair of intracavernous vascular lesions. J Neurosurg. (1983) 58:824–31. 10.3171/jns.1983.58.6.0824 6854374

[4] Dolenc VV Skrap M Sustersic J Skrbec M Morina A. A transcavernous-transsellar approach to the basilar tip aneurysms. Br J Neurosurg. (1987) 1:251–9. 10.3109/02688698709035309 3267289

[5] Dolenc VV. A combined epi- and subdural direct approach to carotid-ophthalmic artery aneurysms. J Neurosurg. (1985) 62:667–72. 10.3171/jns.1985.62.5.0667 3989589

[6] Backes D Rinkel GJ Laban KG Algra A Vergouwen MD. Patient- and aneurysm-specific risk factors for intracranial aneurysm growth: a systematic review and meta-analysis. Stroke. (2016) 47:951–7. 10.1161/STROKEAHA.115.012162 26906920

[7] Can A Mouminah A Ho AL Du R. Effect of vascular anatomy on the formation of basilar tip aneurysms. Neurosurgery. (2015) 76:62–6. 10.1227/NEU.0000000000000564 25255256

[8] Nanda A Sonig A Banerjee AD Javalkar VK. Microsurgical management of basilar artery apex aneurysms: a single surgeon's experience from Louisiana State University, Shreveport. World Neurosurg. (2014) 82:118–29. 10.1016/j.wneu.2013.06.016 23851208

[9] Bohnstedt BN Ziemba-Davis M Sethia R Payner TD DeNardo A Scott J. Comparison of endovascular and microsurgical management of 208 basilar apex aneurysms. J Neurosurg. (2017) 127:1342–52. 10.3171/2016.8.JNS16703 28084909

[10] Dandurand C Prakash S Sepehry AA Tourigny K Haw CS Gooderham P. Basilar apex aneurysm: case series, systematic review, and meta-analysis. World Neurosurg. (2020) 138:e183–90. 10.1016/j.wneu.2020.02.064 32084621

[11] Spiessberger A Strange F Fandino J Marbacher S. Microsurgical clipping of basilar apex aneurysms: a systematic historical review of approaches and their results. World Neurosurg. (2018) 114:305–16. 10.1016/j.wneu.2018.03.141 29602006

[12] Yonekawa Y Khan N Imhof HG Roth P. Basilar bifurcation aneurysms. Lessons learnt from 40 consecutive cases. Acta Neurochir Suppl. (2005) 94:39–44. 10.1007/3-211-27911-3_7 16060239

[13] Al-Khayat H Al-Khayat H White J Manner D Samson D. Upper basilar artery aneurysms: oculomotor outcomes in 163 cases. J Neurosurg. (2005) 102:482–8. 10.3171/jns.2005.102.3.0482 15796383

[14] Chanda A Nanda A. Anatomical study of the orbitozygomatic transsellar-transcavernous-transclinoidal approach to the basilar artery bifurcation. J Neurosurg. (2002) 97:151–60. 10.3171/jns.2002.97.1.0151 12134906

[15] Zhao X Labib MA Shaffer KV Moreira LB Ramanathan D Naeem K. Tailoring the surgical corridor to the basilar apex in the pretemporal transcavernous approach: morphometric analyses of different neurovascular mobilization maneuvers. Acta Neurochir (Wien). (2020) 162:2731–41. 10.1007/s00701-020-04490-8 32757048

[16] Goehre F Lehecka M Jahromi BR Lehto H Kivisaari R Hijazy F. Subtemporal approach to posterior cerebral artery aneurysms. World Neurosurg. (2015) 83:842–51. 10.1016/j.wneu.2015.01.042 25683130

[17] Honda M Tsutsumi K Yokoyama H Yonekura M Nagata I. Aneurysms of the posterior cerebral artery: retrospective review of surgical treatment. Neurol Med Chir. (2004) 44:164–8. 10.2176/nmc.44.164 15185754

[18] Nakagawa H Nakajima S Nakajima Y Furuta Y Nishi O Nishi K. Bilateral oculomotor nerve palsies due to posterior cerebral arterial compression relieved by microvascular decompression–case report. Neurol Med Chir. (1991) 31:45–8. 10.2176/nmc.31.45 1712923

[19] Montemurro N Perrini P Lawton MT. Unsuccessful bypass and trapping of a giant dolichoectatic thrombotic basilar trunk aneurysm. What went wrong? Br J Neurosurg. (2022) 17:1–4. 10.1080/02688697.2022.2077306 35579078

[20] Musara A Yamada Y Takizawa K Seng LB Kawase T Miyatani K. anterior temporal approach and clipping of a high-riding basilar tip aneurysm: case report and review of the surgical technique. Asian J Neurosurg. (2019) 14:1283–7. 10.4103/ajns.AJNS_121_19 31903379 PMC6896634

